# Perinatal Outcomes in Mexican Women with a History of Myomectomy: A Retrospective Cohort Study

**DOI:** 10.3390/jcm14217677

**Published:** 2025-10-29

**Authors:** Fela Vanesa Morales-Hernández, Jocelyn Andrea Almada-Balderrama, Andrea Alicia Olguín-Ortega, Pilar de Abiega-Franyutti, Enrique Reyes-Muñoz, Myrna Souraye Godines-Enriquez

**Affiliations:** 1 Division of Education in Health Sciences, Instituto Nacional de Perinatología Isidro Espinosa de los Reyes, Montes Urales 800, Mexico City 11000, Mexico; vanesa.morales@inper.gob.mx; 2 Department of Reproductive Gynecology, Instituto Nacional de Perinatología Isidro Espinosa de los Reyes, Montes Urales 800, Mexico City 11000, Mexico; jocelyn.almada@gmail.com; 3 Department of Gynecology, Instituto Nacional de Perinatología Isidro Espinosa de los Reyes, Montes Urales 800, Mexico City 11000, Mexico; olguin.andrea@gmail.com; 4 Coordination of Gynecological and Perinatal Endocrinology, Instituto Nacional de Perinatología Isidro Espinosa de los Reyes, Montes Urales 800, Mexico City 11000, Mexico; pilardeabiega@gmail.com (P.d.A.-F.); enrique.reyes@inper.gob.mx (E.R.-M.); 5 Science and Health Faculty, Universidad Anáhuac México, Campus Norte, Huixquilucan 52786, Mexico

**Keywords:** pregnancy outcomes, myomectomy, obstetric hemorrhage, obstetric hysterectomy, uterine rupture

## Abstract

**Background/Objectives**: Myomectomy is the preferred treatment for women with uterine fibroids who desire to preserve their fertility. This study aimed to compare perinatal outcomes between Mexican women with and without a history of myomectomy, matched in a 1:2 ratio based on maternal age and parity. **Methods**: A retrospective cohort study was conducted involving women with and without a history of myomectomy who received prenatal care and delivered at a tertiary care hospital in Mexico City. Women with comorbidities such as pregestational diabetes, chronic hypertension, autoimmune diseases, nephropathy, cardiomyopathy, and cancer were excluded from the study. Group 1 consisted of women with a history of myomectomy, and Group 2 included matched women without such a history. The following perinatal outcomes were evaluated: miscarriage, preterm birth, cesarean section, obstetric hemorrhage, placenta previa, surgical adhesions, and obstetric hysterectomy. Adjusted relative risk (aRR) with 95% confidence intervals (CI) was calculated. **Results**: A total of 122 women were analyzed in group 1, and 244 in group 2. The risk of obstetric hemorrhage aRR 7.5 (95% CI 3.9–11.9), surgical adhesions aRR 11.8 (5.3–20.7), and placenta accreta aRR 15.3 (1.3–111) were significantly higher in Group 1 compared to Group 2. Other outcomes, including miscarriage, preterm birth, cesarean section, placenta previa, and obstetric hysterectomy, were similar between groups. **Conclusions**: Mexican pregnant women with a history of myomectomy have a higher risk of obstetric hemorrhage, surgical adhesions, and placenta accreta compared to those without such a history.

## 1. Introduction

Uterine myomas, also known as fibroids, are the most common benign gynecological tumors found in women [1,2,3]. Their prevalence ranges from 20% to 40% in women of reproductive age and can reach as high as 80% in women over 50 [4,5]. While about 60% are asymptomatic, roughly 30% report abnormal uterine bleeding, 25% experience compression symptoms like pelvic pain and urinary issues, and 15% face infertility challenges [1,6].

Additionally, the presence of fibroids is associated with a higher risk of complications in future pregnancies. These complications may include miscarriage, placenta previa, abnormal fetal positioning, preterm birth, cesarean delivery, and postpartum hemorrhage [7].

Beyond their gynecological effects, fibroids have been associated with adverse pregnancy outcomes such as miscarriage, placenta previa, malpresentation, preterm delivery, cesarean birth, and postpartum hemorrhage [7]. The location of fibroids is clinically important, as submucosal and some intramural types that distort the uterine cavity are most likely to impair fertility and are often considered for surgical removal [8,9]. Treating uterine myomas in women who wish to preserve fertility can be challenging. Interventional options include myomectomy, uterine artery embolization, ultrasound, and medical therapy [10]. Nonetheless, myomectomy remains the preferred treatment for symptomatic women seeking future fertility, as it is consistently associated with higher pregnancy rates and better symptom management [1,11]. This procedure can be done using various approaches, with the choice mainly based on fibroid features and surgical expertise [7]. Minimally invasive techniques, while reducing perioperative complications, may raise concerns about the integrity of the uterine scar. These surgical considerations are especially important because they can affect obstetric safety and perinatal outcomes in future pregnancies [12].

Several studies have reported complications after myomectomy, including surgical adhesions, miscarriage, uterine scar dehiscence, uterine rupture, abnormal placentation, preterm birth, and fetal growth restriction [10,11]. Surgical factors may influence these risks, but current evidence remains insufficient to evaluate them individually [13].

One of the most concerning obstetric complications for women with a history of myomectomy is uterine rupture. The American College of Obstetricians and Gynecologists (ACOG) recommends that pregnant women with a history of myomectomy involving entry into the endometrial cavity should have abdominal deliveries to reduce this risk [14]. Statistics show a 0.47% chance of uterine rupture after myomectomy, whether performed using an open or laparoscopic approach [15]. However, the evidence supporting this recommendation remains limited. Extensive cohort studies indicate that myomectomy is an independent risk factor for uterine rupture, a complication that can severely affect fetal prognosis and lead to poor perinatal outcomes if not managed quickly [16].

Another complication associated with high morbidity and mortality is placenta accreta spectrum (PAS) disorders. Several studies and meta-analyses indicate that myomectomy is associated with an increased risk of PAS, particularly when the uterine cavity is entered during surgery. This contributes substantially to severe maternal and neonatal complications, including increased rates of prematurity and the need for neonatal intensive care unit admission [10,11,17].

Additionally, women with a history of myomectomy face a higher risk of obstetric hemorrhage. Evidence shows increased rates of transfusion, uterotonic use, intestinal injury, and obstetric hysterectomy in this population, with the risk particularly elevated when large fibroids are removed [3,18]. Considering these potential complications, the choice of delivery method remains a contentious issue. Although vaginal delivery after myomectomy can be successful in the majority of cases, population-based data indicate that the overall risk of cesarean delivery is markedly higher among women with a history of this procedure [19,20].

Another aspect of fibroids during pregnancy is when women with fibroids become pregnant, which is important for early pregnancy assessment of the initial size and number of myomas due to the risk of growth, mainly during the first half of pregnancy [21]. In recent years, some biomarkers have been proposed for measurement during the first trimester to predict or correlate with myoma growth [22,23]. Human chorionic gonadotropin (hCG) serves as a biomarker, providing valuable insights into perinatal outcomes. Research indicates that chorionic gonadotropin is involved in regulating prolactin secretion within fibroid cells. This hormonal relationship is significant because prolactin plays a critical role in cellular functions related to growth and proliferation, and the size of myomas is directly correlated with pregnancy complications such as obstetric hemorrhage [24].

Regarding neonatal outcomes, women with a history of myomectomy are at higher risk of preterm birth and delivering low birth weight infants. Some studies also suggest that resection of intramural fibroids may be associated with a greater risk of preterm birth and fetal growth restriction compared with subserosal fibroid removal [3].

However, the available evidence remains limited, deriving mainly from studies conducted in European, North American, and Asian populations. Although large international cohorts demonstrate increased obstetric and perinatal risks after myomectomy, there is a striking absence of data from Latin America, particularly from Mexico. Generating evidence in this context is crucial for adapting perinatal management strategies and providing tailored counseling for affected women. Therefore, the objective of this study was to compare adverse perinatal outcomes in Mexican women with and without a history of myomectomy, matched by maternal age and parity.

## 2. Materials and Methods

### 2.1. Study Design and Participants

This historical cohort study was conducted at a tertiary hospital in Mexico City. It included pregnant women with a history of myomectomy who received prenatal care and delivered at the National Institute of Perinatology (INPer) between January 2018 and December 2023. Each woman with a history of myomectomy was matched with two women without such a history based on maternal age and parity, using a 1:2 ratio. Exclusion criteria for both groups comprised multiple pregnancies, comorbidities such as cardiomyopathy, autoimmune diseases, pregestational diabetes, chronic hypertension, cancer, uterine fibroids, and women who delivered at other institutions. The study adhered to the principles outlined in the Declaration of Helsinki and was approved by the Institutional Review Board, with registration number CEI-RETRO-07-2025. It posed no risk to participants; data were collected retrospectively from electronic records, and participants did not sign an informed consent form.

### 2.2. Data Collection

Data were collected from patient records through the institutional electronic system using keywords like “history of myomectomy” and “pregnancy.” The data included maternal details such as age, infertility history, previous pregnancies, year of myomectomy, size of resected fibroids, entry into the endometrial cavity, type of conception (spontaneous or assisted), gestational age at delivery, delivery method, and pregnancy outcomes. Neonatal records were reviewed for gestational age, weight, height, Apgar scores, and complications. This information helped build the study database. Additionally, medical records were checked for sociodemographic data, prior myomectomies, and medical history potentially affecting perinatal outcomes.

### 2.3. Primary and Secondary Outcomes

The primary aim of this study was to compare the occurrence of APOs in Mexican women with and without a history of myomectomy. The APOs included were miscarriage, defined as the natural ending of a pregnancy before 20 weeks of gestation or when the fetal weight is less than 500 g [25]. Preterm birth was defined as the birth of a baby before 37 weeks of gestation [26]. Obstetric hemorrhage was characterized by an estimated blood loss of more than 500 mL during a vaginal delivery or more than 1000 mL during a cesarean section [27]. Placenta previa refers to the complete or partial coverage of the internal os of the cervix by the placenta [28]. Surgical adhesions are bands of scar-like tissue that develop between two surfaces inside the body, causing them to stick together [29]. An obstetric hysterectomy is a life-saving procedure where the uterus is surgically removed, typically performed in cases of uncontrollable maternal hemorrhage when other conservative management options have failed [30].

### 2.4. Sample Size and Sampling

The sample size calculation aimed to detect a 15% difference in the incidence of obstetric hemorrhage between women with a history of myomectomy (30%) and those without (15%). With a significance level (alpha) of 0.05 and statistical power of 80%, using the program available at https://www.stat.ubc.ca/~rollin/stats/ssize/b2.html, accessed on 3 February 2025, it was determined that at least 121 participants per group are necessary to ensure adequate statistical reliability. 

### 2.5. Statistical Analysis

Statistical analysis was conducted using SPSS software (version 26.0; IBM SPSS Statistics for Windows). The data distribution was evaluated with the Kolmogorov–Smirnov normality test. Chi-square tests were used for categorical variables, while Student’s *t*-tests were applied to continuous variables, which are presented as mean ± standard deviation. A *p*-value of less than 0.05 was considered statistically significant. The relative risk (RR) and 95% confidence intervals (CI) were calculated for each adverse pregnancy outcome (APO). Logistic regression analysis was conducted to identify potential confounders such as body mass index, weeks of gestation at admission, history of infertility, and previous cesarean section, reporting adjusted relative risk (aRR) with 95% CIs for each APO.

## 3. Results

### 3.1. Participants’ Enrollment and Follow-Up

**Figure 1:**
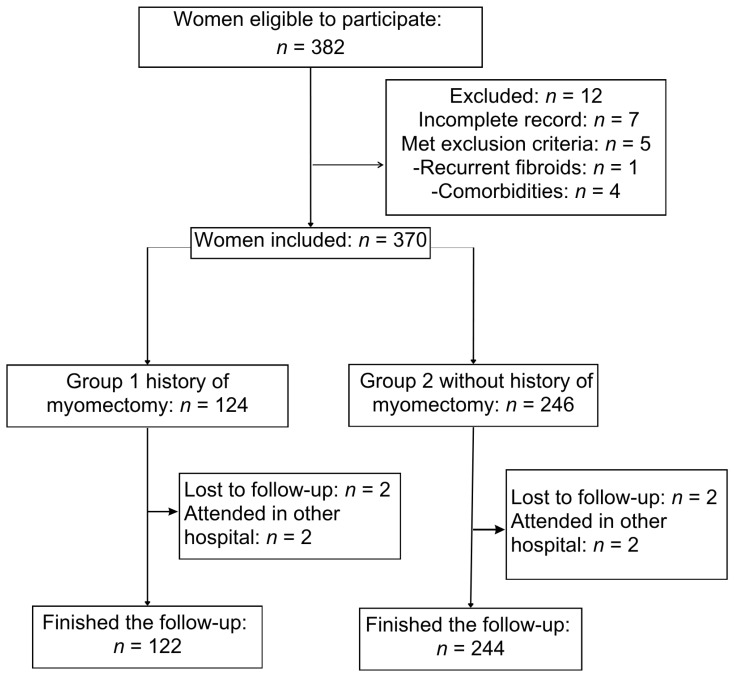
Participant enrollment flow diagram for the historic cohort study.

Figure 1 illustrates the flowchart for the study population. A total of 382 women were identified between January 2018 and December 2023. Twelve women were excluded because they had incomplete records (n = 7) or did not meet the inclusion criteria (n = 5); two in Group 1 (recurrent fibroid and type 2 diabetes) and three in Group 2 (due to chronic hypertension, type 2 diabetes, and systemic lupus erythematosus). A total of 270 women met the inclusion criteria, comprising 122 women in Group 1 and 244 women in Group 2 who completed the follow-up.

### 3.2. Demographic and Clinical Characteristics

Women with a history of myomectomy had similar average maternal ages and number of previous pregnancies. However, Group 1 had a significantly lower pregestational body mass index (BMI) (27.8 vs. 29.02, *p* = 0.02) and lower rates of prior cesarean deliveries (19.6% vs. 31.9%, *p* = 0.01). The gestational age at the start of prenatal care was significantly earlier in Group 1 compared to Group 2. The proportion of women with normal weight was significantly higher in Group 1, while there were no differences in the rates of overweight and obesity between groups. Additionally, a history of infertility was much more common in Group 1 (48% vs. 8.2%, *p* < 0.001), with primary infertility accounting for 31% of cases in Group 1 versus 3.7% in Group 2 (*p* < 0.001) (see Table 1).

### 3.3. Surgical Management of Myomectomy

Table 2 shows the characteristics of fibroid surgical management and modes of conception in women with a history of myomectomy. Myomectomy was performed at the INPer in 60.7% (n = 74) of cases and at other institutions in 39.3%. Laparotomy was the most common surgical approach, followed by laparoscopy and hysteroscopy. Regarding the number of resected fibroids, 43.4% had one fibroid removed, 23.8% had two, 11.5% had three, 9% had four, and 12.3% had five or more fibroids. In 25.4% of cases, the surgeon entered the endometrial cavity. Fibroids larger than 5 cm were resected in 52.5% of women, although this information was unknown for 34.4% of cases. In Group 1, conception among women with infertility occurred spontaneously in 77.9% of pregnancies, while 20.3% required in vitro fertilization.

### 3.4. Maternal and Neonatal Outcomes

Perinatal outcomes are summarized in Table 3. The average gestational age at delivery, neonatal birth weight, and Apgar scores < 8 were similar between groups. Additionally, the incidence and risks of miscarriage, preterm birth, and cesarean delivery were comparable across the groups.

The adjusted relative risk indicated that women with a history of myomectomy had a higher risk of intra-abdominal surgical adhesions, obstetric hemorrhage, and placenta accreta. The four cases of placenta accreta were diagnosed antenatally; two were located in the anterior uterine wall and two in the posterior uterine wall. Other complications, such as placenta previa, uterine rupture, miscarriage, preterm birth, cesarean delivery, hysterectomy, vesical injury, abruptio placentae, and neonates small for gestational age, were similar between the groups (Table 3).

## 4. Discussion

### 4.1. Principal Findings

This study found that Mexican pregnant women with a history of myomectomy have a higher risk of obstetric hemorrhage, surgical adhesions, and placenta accreta compared to those without such a history. Notably, women who had a previous myomectomy experienced higher rates of infertility than those who did not undergo the procedure; however, the most common mode of conception among women with myomectomy was spontaneous, without assisted reproductive techniques.

### 4.2. Comparison with Existing Literature

Multiple studies have shown that uterine fibroids can negatively impact fertility by causing anatomical changes that obstruct fertilization and implantation [21,31,32]. The biological plausibility of these links has been related to hormonal and vascular mechanisms that influence fibroid growth during pregnancy [21,24]. Among hormonal mediators, hCG plays a key role, stimulating proliferation both directly through hCG receptors on leiomyomas and indirectly via prolactin-mediated pathways. Previous evidence strongly supports this link between early pregnancy hCG levels and fibroid growth, with coefficients reaching up to R = 0.69 (*p* < 0.0001) [22].

Myomectomy, however, has been linked to higher conception rates and lower miscarriage rates, with post-myomectomy pregnancy rates reaching up to 75.6% in infertile women when other factors are controlled for [33]. Although this research was not limited to infertile women, our findings align with this literature: 77% of the 59 women with infertility who had a myomectomy conceived spontaneously afterward. This underscores that spontaneous conception can still occur after myomectomy, an important point to consider when counseling patients about fertility prospects. 

Obstetric outcomes after myomectomy are a concern due to potential endometrial damage from hysteroscopic procedures or uterine cavity entry during laparotomy or laparoscopy, as well as scarring-related changes in myometrial integrity [34]. A key finding of our study is the increased risk of obstetric hemorrhage at delivery among women with a history of myomectomy (29.5% vs. 12.5%), in line with prior reports [11]. The biological plausibility of this finding relates to impaired contractility in scarred myometrium and abnormal vascularization at previous surgical sites [20]. This highlights the importance of preparing for hemorrhage during labor and ensuring access to blood products, uterotonics, and multidisciplinary support.

Placenta accreta spectrum disorders are increasingly common and can be life-threatening. The association between myomectomy and abnormal placental attachment remains debated [11,35]. A recent cohort study of over one million women found an incidence of 0.96% for PAS disorders in women with previous myomectomy, with an odds ratio (OR) of 2.28 (95% CI: 1.85–2.81). The incidence was higher in women who had hysteroscopic myomectomy compared to other surgical methods [36]. In our study, women with a history of myomectomy had a higher risk of placenta accreta (3.3%) compared to those without such a history (0%). The four cases of placenta accreta diagnosed were identified during prenatal assessment; two were on the anterior uterine wall and two on the posterior wall. However, the records did not specify whether these placenta accreta cases were related to myomectomy scars. 

Historically, the risk of uterine rupture after myomectomy was reported as 2.5% [37]. Recent research, however, shows this risk has dropped to 0.79% [13,15]. Evidence indicates a higher risk following laparoscopic myomectomy compared to abdominal myomectomy (1.2% vs. 0.4%), influenced by factors such as thermal damage, the number of suture layers, and suturing techniques. In our study, there was one case of uterine rupture after laparoscopic myomectomy. Despite this, the risk remains lower than the 1% risk associated with cesarean delivery, suggesting that rupture risk after myomectomy is low in women without cavity entry, making vaginal delivery a safe option [13]. Though rare, uterine rupture can have catastrophic consequences for fetal outcomes. The low occurrence in our cohort supports the safety of vaginal delivery when the uterine cavity has not been entered but also highlights the importance of close intrapartum monitoring.

This study found that women with a history of myomectomy have a higher risk of bladder injury, surgical adhesions, and obstetric hysterectomy, consistent with findings from previous research [11]. These complications reflect the increased surgical complexity of deliveries after myomectomy. Recognizing this risk is essential for surgical planning, emphasizing the importance of experienced teams and multidisciplinary support.

Additionally, the literature suggests that these women may face a higher risk of gestational hypertensive diseases; however, this was not observed in our study. In 2024, Chen et al., published an analysis showing that a time interval from myomectomy to pregnancy of less than 6 months or 12 months or more is associated with higher risks of gestational hypertensive disorders compared to a time interval of 6 to 11 months, especially for women over 35 years old [33]. We did not evaluate the time interval between myomectomy and pregnancy as a potential risk factor for gestational hypertensive disorders, but it may be an important area for future research.

Regarding the mode of delivery, available evidence suggests that women with a history of myomectomy are at a higher risk of cesarean delivery, regardless of whether the cavity entry occurred [37,38,39]. However, our study did not observe such differences. This discrepancy may stem from variations in sample size, surgical technique, and population characteristics.

Existing evidence indicates that women with a history of myomectomy are at higher risk of preterm birth [38,39]. However, this study differs from those findings, as no differences were observed in gestational age at delivery or neonatal morbidity.

On the other hand, a systematic review and meta-analyses have concluded that uterine fibroids during pregnancy have been associated with a higher risk of preterm birth, threatened preterm labor, preterm premature rupture of membranes, fetal malpresentation, placental abruption, lower gestational age and birthweight at delivery, and a higher cesarean delivery rate [40,41].

Hypothetically, the faster a fibroid grows—and thus enlarges—the higher the chance it may outgrow its blood supply, causing necrosis [42]. Necrosis typically triggers inflammation through various cellular and molecular mechanisms. The protein high mobility group box 1 (HMGB 1) plays a crucial role in sterile inflammation after injury by recruiting neutrophils and promoting necrosis. It is passively released during necrosis, stimulating inflammation by increasing tumor necrosis factor alpha (TNF-α) production [42]. Besides HMGB 1, necrotic cells activate the nuclear factor kappa B (NF-κB) pathway through the secretion of heat shock proteins (HSPs). When extracellular, HSPs specifically trigger the NF-κB pathway, a response associated with necrosis rather than apoptosis. Activation of NF-κB leads to increased transcription of inflammation-related genes such as IL-1 β, IL-8, TNF-α, and COX-2. Consequently, necrosis in fibroids can start an inflammatory cascade that may contribute to spontaneous preterm birth [42].

### 4.3. Strengths and Limitations

This study is the first to report perinatal outcomes among Mexican women with a history of myomectomy compared to those without. Its key strength is assessing the surgical approach, along with the number, size, and location of fibroids, as well as cavity entry. However, limitations include its retrospective design, being conducted at a single center, and a sample size that limits the evaluation of secondary outcomes. Moreover, data collection was challenging due to missing information about women who had myomectomies elsewhere. The study also did not examine other comorbidities that could affect perinatal outcomes, highlighting the need for further research to address these issues.

### 4.4. Clinical Implications

Given the observed rate of obstetric hemorrhage in our study population, we recommend that blood components be routinely prepared before delivery for women with a history of myomectomy involving fibroids larger than five centimeters. Our findings highlight the importance of gynecologists and obstetricians counseling women about the maternal risks associated with delivery after a prior myomectomy. Additionally, this study offers clinically relevant information for physicians regarding the need to request blood components before delivery for this patient group.

Further research is needed using biomarkers in early pregnancy, such as chorionic gonadotropin, metalloproteinases, and HMGB-1 [21,22,23,24,42,43], to assess their potential for predicting women with uterine fibroids who are at higher risk of faster growth and adverse perinatal outcomes.

## 5. Conclusions

Women with a history of myomectomy face a higher risk of adverse perinatal outcomes, including increased risk of obstetric hemorrhage, abnormal placental insertion, and surgical adhesions, compared to women without a history of myomectomy.

## Figures and Tables

**Table 1:** Clinical characteristics in pregnant women with and without a history of myomectomy.

Characteristic	Group 1, Women with a History of Myomectomy (n = 122)	Group 2, Women Without a History of Myomectomy (n = 244)	*p*
Age	36.2 ± 5.2	35.8 ± 5.4	0.49
Pregestational BMI	27.8 ± 4.2	29.02 ± 4.7	0.02
Gestational age at admission	11.6 ± 5.1	29.0 ± 7.1	0.0001
Gravidity			
0	44 (36.1)	72 (29.5)	0.25
1	43 (32.5)	103 (42.2)	0.24
≥2	35 (28.6)	69 (28.2)	0.96
Normal weight (BMI 18.5−24.99 kg/m^2^)	29 (23.8)	35 (14.3)	0.03
Overweight (BMI 25−29.99 Kg/m^2^)	59 (48.4)	120 (49.2)	0.97
Obesity (BMI ≥ 30 kg/m^2^)	34 (27.9)	89 (36.5)	0.12
Previous cesareans			
1	24 (19.6)	78 (31.9)	0.01
2	4 (3.3)	6 (2.5)	0.91
≥3	1 (0.8)	0 (0)	0.98
History of infertility	59 (48%)	20 (8.2%)	0.0001
Primary infertility	38 (31%)	9 (3.7%)	0.0001
Secondary infertility	21 (17%)	11 (4.5%)	0.0001

BMI = Body Mass Index. Value expressed as mean and standard deviation or frequency and percentage.

**Table 2:** Characteristics of fibroid surgical management and mode of conception in women with prior myomectomy.

Characteristics	Women with Prior Myomectomy n = 122
Laparotomy	87 (71.3)
Laparoscopy	26 (21.3)
Hysteroscopy	9 (7.4)
Entry into the endometrial cavity	
Yes	31 (25.4)
No	81 (66.4)
Unknown	10 (8.2)
Size of the largest resected myoma	
>5 cm	64 (52.5)
3–5 cm	15 (12.3)
<3 cm	2 (1.6)
Unknown fibroid size	41 (33.6)
Number of resected myomas	
1	53 (43.4)
2	29 (23.8)
3	14 (11.5)
4	11 (9.0)
≥5	15 (12.3)
Modes of conception in women with infertility	n = 59
Spontaneous pregnancy	46 (77.9)
Intrauterine insemination	1 (1.7)
In vitro fertilization	12 (20.3)

Values expressed as frequency and percentages.

**Table 3:** Maternal and perinatal outcomes by study group.

Perinatal Outcomes	Total (n = 366)	Group 1 Women with a History of Myomectomy (n = 122)	Group 2 Women Without a History of Myomectomy (n = 244)	*p*	Adjusted * Relative Risk (95% CI)
Weeks of gestation at resolution	38.2 ± 2.3	38.2 ± 2.1	38.2 ± 2.2	0.78	-
Neonatal birth weight (g)	2924.6 ± 572	2931 ± 549	2875 ± 551	0.37	-
Neonatal size (cm)	48.51 ± 2.66	48.4 ± 2.6	48.2 ± 3.1	0.57	-
Apgar < 8	31 (8.5)	14 (12.7)	17 (7.2)	0.19	1.78 (0.72–3.9)
Miscarriage	14 (3.8)	6 (4.9)	8 (3.3)	0.31	1.83 (0.55–5.5)
Preterm birth	47 (12.8)	16 (13.1)	31 (12.7)	0.74	1.12 (0.55–2.1)
Cesarean delivery	317 (85.2)	109 (89.3)	208 (85.2)	0.15	1.06 (0.96–1.12)
Neonates small for gestational age	47 (12.8)	14 (11.5)	33 (13.5)	0.78	0.89 (0.4–1.8)
Surgical adhesions	44 (12.0)	37 (30.3)	7 (2.8)	0.0001	11.8 (5.3–20.7)
Obstetric hemorrhage	52 (14.2)	39 (32)	12 (4.9)	0.0001	7.5 (3.9–11.9)
Placenta accreta	4 (1.1)	4 (3.3)	0 (0)	0.03	15.3 (1.3–111)
Placenta previa	3 (0.82)	2 (1.64)	1 (0.4)	0.19	7.2 (0.35–95.9)
Hysterectomy	3 (0.82)	3 (2.46)	0 (0)	0.09	11.3 (0.69–111)
Vesical injury	2 (0.55)	2 (1.6)	0 (0)	0.87	1.5 (0.11–115)
Abruptio placentae	4 (1.1)	2 (1.64)	3 (1.2)	0.38	2.8 (0.26–22.9)
Preeclampsia	11 (3.0)	3 (2.5)	8 (3.3)	0.58	0.60 (0.93–3.46)
Uterine rupture	1 (0.27)	1 (0.82)	0 (0)	0.55	5.7 (0.45–91.6)

* Adjusted for body mass index, weeks of gestation at admission, history of infertility, and previous cesarean section.

## Data Availability

Data supporting the reported results are in the article and can be obtained by request to the corresponding author.

## Abbreviations

The following abbreviations are used in this manuscript:

ACOG	American College of Obstetricians and Gynecologists
hCG	Human Chorionic Gonadotropin
INPer	Instituto Nacional de Perinatologia Isidro Espinosa de los Reyes
PAS	Placenta accreta spectrum
HMGB 1	High Mobility Group Box 1
TNF-α	Tumor Necrosis Factor Alpha
HSPs	Heat Shock Proteins
